# Supplementary material for: Continuous Production of Highly Tuned Silk/Calcium-Based Composites: Exploring New Pathways for Skin Regeneration
Source: Molecules. 2022 Mar 30;27(7):2249. doi: 10.3390/molecules27072249 (PMC9000890; doi:10.3390/molecules27072249)
Supplement: Supplementary file 1 [file molecules-27-02249-s001.zip › molecules-1611644-supplementary.pdf]

Supplementary Material

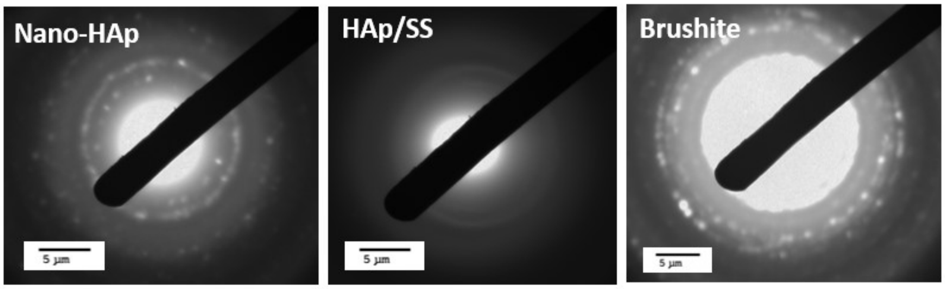

Figure S1. SAED (Selected area diffraction) (TEM JEOL, 2200 FS / EDS Oxford, INCA Energy Table 250. analysis on nano-HAp, HAp/SS and Brushite particles. Note: the size of the SAED rings is proportional to the sample's crystallinity.
